# Myeloid-Derived Suppressor Cells as Target of Phosphodiesterase-5 Inhibitors in Host-Directed Therapeutics for Tuberculosis

**DOI:** 10.3389/fimmu.2020.00451

**Published:** 2020-03-25

**Authors:** Vinzeigh Leukes, Gerhard Walzl, Nelita du Plessis

**Affiliations:** Division of Molecular Biology and Human Genetics, Faculty of Medicine and Health Sciences, DST-NRF Centre of Excellence for Biomedical Tuberculosis Research, South African Medical Research Council for Tuberculosis Research, Stellenbosch University, Cape Town, South Africa

**Keywords:** tuberculosis, host-directed therapy, myeloid-derived suppressor cells, sildenafil, phosphodiesterase-5 inhibitors

## Abstract

Resistance toward current and new classes of anti-tuberculosis (anti-TB) antibiotics are rapidly emerging; thus, innovative therapies focused on host processes, termed host-directed therapies (HDTs), are promising novel approaches for shortening therapy regimens without inducing drug resistance. Development of new TB drugs is lengthy and expensive, and success is not guaranteed; thus, alternatives are needed. Repurposed drugs have already passed Food and Drug Administration (FDA) as well as European Medicines Agency (EMA) safety requirements and may only need to prove efficacy against *Mycobacterium tuberculosis* (*M.tb*). Phosphodiesterases (PDEs) hydrolyze the catalytic breakdown of both cyclic adenosine monophosphate (cAMP) and cyclic guanosine monophosphate (cGMP) to their inactive mononucleotides. Advances in molecular pharmacology have identified 11 PDE families; and the success of sildenafil, a PDE-5 selective inhibitor (PDE-5i), in treating pulmonary hypertension and erectile dysfunction has invigorated research into the therapeutic potential of selective PDE inhibitors in other conditions. Myeloid-derived suppressor cells (MDSCs) suppress anti-TB T-cell responses, likely contributing to TB disease progression. PDE-5i increases cGMP within MDSC resulting in the downregulation of arginase-1 (ARG1) and nitric oxide synthase 2 (NOS2), reducing MDSC's suppressive potential. The effect of this reduction decreases MDSC-induced T-cell-suppressive mechanisms. This review highlights the possibility of HDT targeting of MDSC, using a PDE-5i in combination with the current TB regimen, resulting in improved TB treatment efficacy.

## Tuberculosis Treatment Challenges

Tuberculosis (TB), caused by *Mycobacterium tuberculosis* (*M.tb*), is among the top 10 causes of death worldwide (1). The current 6-month regimen for drug-sensitive TB only achieves an 82% success rate after strict adherence, leaving 1.1 million people sick (1). Increasing resistance to anti-TB drugs compounded with time-consuming and costly drug development have further hampered treatment. More effective, cheaper, and smarter drug discovery approaches represent promising solutions to these challenges. Drug repurposing, new indications for existing drugs, is based on poly-pharmacology principles whereby one drug is able to act on multiple targets or disease pathways (2). Drug repurposing has been accepted globally as being more rapid and cost-effective than have traditional drug discovery approaches (3, 4). Several research groups have recently published their research and novel ideas on the introduction of HDT in the treatment of TB. Zumla et al. made the case for parallel investments into HDT in TB, specifically those with the potential to shorten the duration of TB therapy and improve treatment outcomes for drug-susceptible and drug-resistant *M.tb* strains (5). Sachan et al. (6) argued that supplementing anti-TB therapy with host response modulators will overcome antibiotic resistance and will aid in killing non-replicating bacilli. Sachan et al. also make the case that deployment of HDT in TB may be optimally achieved through macrophage-targeted inhaled delivery systems (6). Furthermore, recent studies have illustrated multiple potential host therapeutic targets against *M.tb*. These include targeting granuloma structure (enbrel and bevacizumab), autophagy induction (vitamin D3, rapamycin, and carbamazepine), anti-inflammatory response (ibuprofen, zileuton, prednisone, sildenafil, and doxycycline), cell-mediated immune response (statin, metformin, and ipilimumab), and anti-*M.tb* monoclonal antibodies (anti-LAM monoclonal IgG3/IgA/IgM) (7). Most recently, a comprehensive review focusing on HDT strategies to improve treatment outcome in TB highlighted preclinical studies that aimed to enhance endogenous pathways and/or limit destructive host responses. It discussed promising preclinical candidates and forerunning compounds at advanced stages of clinical investigation in TB HDT efficacy trials (8). Moreover, the National Institutes of Health (NIH) hosts a resource database (clinicaltrials.gov) of privately and publicly funded human clinical trials investigations on adjunct therapies for various forms of TB. Taken together, the development of repurposed drugs as adjunct anti-TB therapies is being actively pursued, as they would have a positive impact on treatment success rates globally.

### Inadequate Immune Response

The inadequacy of immune response balance to the *M.tb* pathogen results in excessive pro-inflammatory processes resulting in severe tissue damage in the lungs (9). This tissue damage is required for bacterial spread, as pathogen entry into pulmonary airways permits aerosol transmission. Recent studies have also highlighted that the immune pathology of TB patients is further affected by a balance of both pathogen- and host-induced signaling events (10, 11). Agarwal et al. showed that among 17 adenylate cyclase genes present in *M.tb*, Rv0386 is required for virulence. They demonstrated that it facilitates delivery of bacterial-derived cAMP into the macrophage cytoplasm, enabling *M.tb* to modify both its intracellular and tissue environments to facilitate long-term survival (10). *M.tb* is also capable of releasing and trafficking bioactive lipids to exacerbate infection pathology and drive granuloma progression leading to caseation and spread (11). Modulation of host response by repurposed drugs in combination with anti-TB drugs during *M.tb* infection represents an adjunctive treatment approach to improve current treatment efficacy.

## Cyclic Adenosine Monophosphate and *Mycobacterium tuberculosis*

cAMP is an important second messenger signaling molecule involved in the regulation of many cellular processes during *M.tb* infection (10). *M.tb* manipulates and subverts cAMP signaling pathways within infected host phagocytes, directly influencing bacterial survival in mice (10). Upon infection, *M.tb* produces a burst of cAMP within macrophages. Bacterial-derived cAMP is delivered to the macrophage cytoplasm through expression of a microbial adenylate cyclase gene, resulting in increased cytosolic cAMP levels. This 3–5-fold increase in cAMP concentration compared with baseline triggers the PKA–CREB pathway to upregulate NFκB transcription. Tumor necrosis factor alpha (TNF-α) secretion is elevated as a consequence of bacterial-mediated cAMP signaling subversion during early infection. This fosters bacterial survival by promoting necrosis and granuloma formation (10). Cyclic-di-adenosine monophosphate (c-di-AMP), a double-edged sword, *M.tb*-derived secondary messenger, interferes with host immune signaling pathways (12). Through STING-IRF3 signaling pathway, c-di-AMP induces type I interferon, benefiting the microbe through enhanced immunopathology (13). But c-di-AMP also enhances bacterial killing and autophagy (13). c-di-AMP is recognized *via* macrophage cytosolic surveillance pathways as a pathogen-associated molecular pattern (PAMP). Microbial c-di-AMP production benefits the host by stimulation of autophagy as previously indicated in animal models where *M.tb* expressing excess c-di-AMP displayed loss of pathogenicity (12). Taken together, targeting the network of cAMP-mediated signaling pathways with repurposed drugs could reduce bacterial survival during *M.tb* infection.

## Phosphodiesterase Inhibitors

Inflammation can also be controlled by regulating the activity of phosphodiesterases (PDEs), a group of enzymes that hydrolyze cyclic adenosine and guanosine monophosphates to AMP and GMP (14). Eleven classes of PDEs have been identified in mammals, and inhibitors are available for types 1–5 (15). PDE types 1–3 are able to hydrolyze both cAMP and cGMP, whereas type 4 and type 5 PDEs specifically hydrolyze cAMP and cGMP, respectively. Each PDE type has unique localization and expression profiles, in addition to their differing substrate specificities (16). PDE inhibitors (PDE-i) have become important drugs in human medicine, as they increase cytosolic concentrations of cyclic nucleotides, by inhibiting their breakdown by PDEs. PDE-3i has been used medically to treat intermittent claudication, PDE-4i for chronic obstructive pulmonary disease, and PDE-5i for erectile dysfunction and pulmonary hypertension. In various models of TB disease, PDE-I has shown success as an adjunctive treatment agent (17, 18). In an 8-week mouse model, roflumilast, an FDA-approved PDE-4i, augmented isoniazid action (19). Further investigation into the exact mechanism of PDE-i success in TB mouse models remains to be determined. Sildenafil, an FDA/EMA approved PDE-5i, also known as Viagra®, has been used clinically to treat pulmonary hypertension, cardiac hypertrophy, and erectile dysfunction by increasing intracellular concentrations of cyclic guanosine monophosphate (cGMP) (20, 21). PDE-5i has also shown restorative immune effects and consistent benefits in the treatment of male genitourinary dysfunctions [including benign prostatic hyperplasia (22, 23), lower urinary tract symptoms (24), and Peyronie's disease (25)], as well as neurologic dysfunctions [neurogenesis and recovery from stroke (26–31)], tissue and organ protection [antineoplastic agent (32) and gastrointestinal damage (33, 34)], cutaneous ulcerations [antiphospholipid syndrome (35), scleroderma (36, 37), and systemic sclerosis (38, 39)], transplant and reconstructive surgery (40–44), female genital dysfunctions [fertility and preeclampsia (45–50)], and diabetes [neuropathy and vasculopathy (51–53)]. In oncology, PDE-5 inhibition was tested in mice and shown to be immune restorative by reversing tumor-induced immunosuppression and inducing antitumor immunity that delayed tumor progression. In particular, sildenafil has shown to improve cancer therapy by upregulating T-cell numbers in tumors and increasing T-cell activation and T-cell interleukin (IL)-2 production (54). Subsequently, PDE-5i is being repurposed and tested in human clinical trials for treatment of malignancies.

Because of PDE-5i's success in oncology, this was attempted in TB owing to the long-term chronic inflammatory state common to both diseases. PDE-5i has shown promise in laboratory models of *M.tb* infection/TB disease; however, the effect of PDE-5i on host immune responses, specifically MDSC levels and function, in the context of human TB remains unknown. Reports show that sildenafil addition to standard TB therapy accelerated *M.tb* sterilization in the mouse lung by 1 month as compared with standard treatment alone (55). Thus, adjunct PDE-i together with anti-TB chemotherapy may help shorten treatment duration and improve treatment outcome.

## Sildenafil

Sildenafil has been well-characterized and has known PK, PD, and safety profiles. Sildenafil is rapidly absorbed; acts within 30 min to 1 h; has a short plasma half-life of 4 h; and is well-tolerated in the dosage range of 25 and 100 mg (56, 57). Sildenafil also has a calculated bioavailability of 41% (58). Testing of drug–drug interactions between TB medication and sildenafil would be prudent. Moreover, both sildenafil and first-line TB drugs (isoniazid, rifampicin, pyrazinamide, ethambutol, and rifabutin) share interactions with cytochrome P450 (Cyp3A) (59, 60). In a study by Maiga et al. (55), they utilized cilostazol and sildenafil, both FDA approved, in combination with rifampin in their *in vivo* experiments, with the rationale being that the same compounds could be tested in humans. They concluded that cilostazol does not reduce the efficacy of rifampin, but this remains to be tested for sildenafil (55). Dash et al. (61) used computer modeling studies to examine the docking ability of sildenafil on *M.tb*. They found that according to the “TB-drugome,” the Rv1555 protein is “druggable” with sildenafil and has the potential to inhibit the electron transport function during anaerobic respiration, but further validation with *M.tb* strains is required to provide more accurate and reliable proof. Conclusive evidence showing no reduction in the efficacy of TB medication in the presence of sildenafil is still required and should be investigated. Because coinfection between *M.tb* and HIV is a significant problem worldwide, particularly in South Africa, the interactions between sildenafil and antiretrovirals should also be considered. Sildenafil has been shown to have no significant change on the effect of ARV levels (saquinavir and ritonavir) (62). Sildenafil could therefore be considered for patients with *M.tb* and HIV coinfection.

## Myeloid-Derived Suppressor Cells

MDSCs are a heterogeneous population of myeloid cells, at various stages of differentiation, consisting of immature myeloid cells and also further differentiated early granulocytic or monocytic cells, with the capacity to suppress T-cell functions (63).

MDSC can be divided into two subsets with distinct morphology and suppressive mechanisms: firstly, monocytic MDSC (M-MDSC), morphologically similar to monocytes, macrophages, and dendritic cells, expressing high levels of NO; and secondly, polymorphonuclear MDSC (PMN-MDSC), morphologically similar to granulocytes, expressing high levels of ROS (63, 64). MDSC frequencies are increased in humans at TB diagnosis (65). Others have also found that the predominant subset of cells accumulating in the lungs of mice infected with *M.tb* was Gr1+ (66). This finding is consistent with that of Tsiganov et al., who found an association between TB progression and cells expressing Gr-1 and Ly-6G (67). MDSC with immuno-modulatory and suppressive effects were also significantly increased in the blood and lung components of TB patients (68). While Obregón-Henao et al. (66) showed that Gr1+ MDSC from TB patients highly expressed arginase-1 (ARG1), an immuno-modulatory enzyme that depletes l-arginine, imparting potent immunosuppressive effects on T-cell function. Tsiganov et al. (67) and Daker et al. (68) showed that MDSC suppressed T-cell proliferation in mycobacterial infections *in vitro via* a nitric oxide (NO)-dependent method. Knaul et al. (69) were also consistent in showing accumulation of MDSC during TB but went further to show that these cells, in addition to their immunosuppressive capacity, could phagocytose both BCG and H37Rv. This work suggests that MDSC has a dual role in TB disease: firstly by suppressing T-cell function and secondly harboring *M.tb*. Considering the immunosuppressive properties of MDSC in TB, ablation of these cells represents a feasible target for investigation of potential HDT.

## Myeloid-Derived Suppressor Cell and Phosphodiesterase-5 Selective Inhibitor

**Figure 1:**
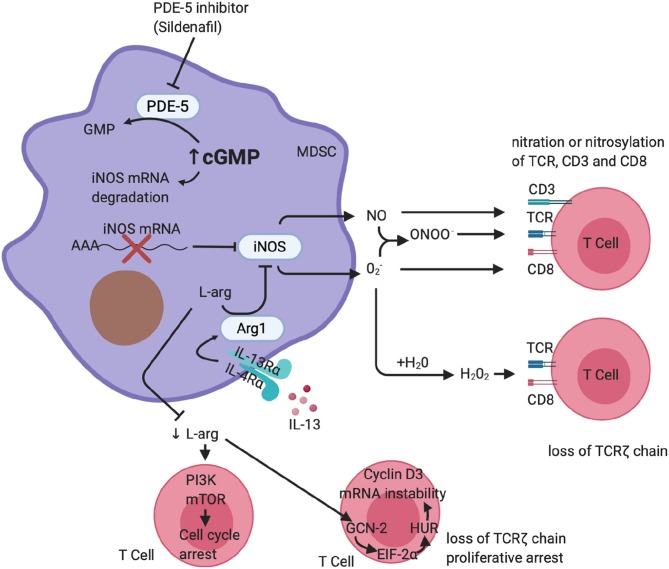
Phosphodiesterase-5 inhibition of MDSC function. A schematic representation of PDE-5 inhibition on MDSC function. PDE-i increases cGMP, which results in destabilization of the iNOS mRNA, reduced synthesis of iNOS, and ultimately less production of NO. It is also able to downregulate the expression of IL4-Rα, resulting in a reduction of arginase-1 expression. This reduces MDSC-mediated suppression of T cells carried out *via* arginase-1 and iNOS. (Figure created using Biorender.com).

Sildenafil downregulates MDSC immunosuppressive activity in cancer. Serafini et al. have shown that sildenafil downregulated MDSC in a mouse model and thereby restored antitumor immunity (54). Sildenafil-mediated downregulation of MDSC resulted in T- and B-cell-dependent immune enhancement and also greatly increased CD8 T-cell recruitment to the inflammation site. The mechanism by which PDE-5 inhibition downregulates MDSC activity is through inhibition of ARG1 and NOS2 expression (Figure 1), which have been shown to be critical in immune suppression (54). More recently, studies have shown an increase of MDSC in melanoma lesions, with an associated downregulation in T-cell activity (70, 71). Pharmacological inhibition of PDE-5 attenuated MDSC immunosuppressive function and significantly increased survival of tumor-bearing mice (70, 71). A case report described a patient with multiple myeloma who, after being treated with a PDE-5 inhibitor, experienced a durable anti-tumor immune response and clinical improvement from reduced MDSC function (72). In the context of pulmonary TB, PDE-i has shown to decrease TB disease severity, pathology, and bacillary load in mouse models, but their effect on host immunity during human *M.tb* infection and TB disease remains poorly defined (17–19, 73).

## Conclusion

The demonstration of a biologically significant role of MDSC during *M.tb* infection would have important implications for clinical studies on TB in South Africa and across the world. Theoretically, drugs that could target MDSC directly or its associated mechanisms could prevent MDSC accumulation and function, potentially overcoming MDSC-mediated immune suppression in TB. These would include medications that would induce differentiation of MDSC into mature non-suppressive cells, inhibit MDSC expansion from hematopoietic precursors, and block MDSC signaling pathways. These strategies are currently explored in mouse models in ongoing clinical trials testing modulation of MDSC by pharmacological intervention in cancer patients, focusing on sildenafil, a substance limiting MDSC immunosuppressive function. PDE-5 inhibitors seem promising in TB models, but the mechanism of PDE-5i improving host *Mtb* control has not been established. Neither has the role of sildenafil on human MDSC in the context of TB been evaluated. Further groundwork is needed to better understand how PDE-5i might be beneficial in combination with TB treatment. Thus, examining the potential role of PDE-5i-mediated MDSC modulation and resultant restored T-cell function in the presence of MDSC should be investigated in the context of TB disease. These investigations will pave the way toward a better understanding of the basic mechanisms of host immune defense and the human capacity to develop immune responses to these infections. In conclusion, data from future studies could serve as the required scientific evidence for clinical investigations using sildenafil administration as adjunct therapy in TB.

## Author Contributions

VL wrote the manuscript, with development contributions by GW and NP.

### Conflict of Interest

The authors declare that the research was conducted in the absence of any commercial or financial relationships that could be construed as a potential conflict of interest.

## Abbreviations

TB: Tuberculosis
HDT: Host-directed therapies
FDA: Food and Drug Administration
EMA: European Medicines Agency
*M.tb*: *Mycobacterium tuberculosis*
PDE: Phosphodiesterase
cAMP: Cyclic adenosine monophosphate
cGMP: Cyclic guanosine monophosphate
MDSC: Myeloid-derived suppressor cell
ARG1: Arginase 1
NOS2: Nitric oxide synthase 2
PKA: Protein kinase A
CREB: Cyclic-AMP response element-binding protein
NFκB: Nuclear factor kappa-light-chain-enhancer of activated B cells
TNF-α: Tumor necrosis factor alpha
c-di-AMP: Cyclic di-AMP
STING: Stimulator of interferon genes
IRF3: Interferon regulator factor 3
PAMP: Pathogen-associated molecular pattern
PDE-i: Phosphodiesterase inhibitor
IL: Interleukin
PK: Pharmacokinetics
PD: Pharmacodynamics
Gr-1: Protein gamma response 1
Ly-6G: Lymphocyte antigen 6 complex locus G6D
NO: Nitric oxide
BCG: Bacillus Calmette–Guérin
VEGF: Vascular endothelial growth factor
GM-CSF: Granulocyte-macrophage colony-stimulating factor
SCF: Stem cell factor
c-kitP: Phosphorylated c-kit
mRCC: Metastatic renal cell carcinoma
Treg: Regulatory T cell
MMP: Matrix metalloproteinase
ATRA: All trans retinoic acid
HIV: Human immunodeficiency virus
ARV: Antiretroviral.
